# Phylogenetic analysis of Harmonin homology domains

**DOI:** 10.1186/s12859-021-04116-5

**Published:** 2021-04-14

**Authors:** Baptiste Colcombet-Cazenave, Karen Druart, Crystel Bonnet, Christine Petit, Olivier Spérandio, Julien Guglielmini, Nicolas Wolff

**Affiliations:** 1 Unité Récepteurs-Canaux, Institut Pasteur, 75015 Paris, France; 2 Collège Doctoral, Sorbonne Université, 75005 Paris, France; 3 Unité de Bio-Informatique Structurale, Institut Pasteur, 75015 Paris, France; 4 Unité de Génétique et Physiologie de l’Audition, Institut Pasteur, 75015 Paris, France; 5 INSERM, Institut de l’Audition, Institut Pasteur, 75012 Paris, France; 6 Hub de Bioinformatique et Biostatistique – Département Biologie Computationnelle, USR 3756 CNRS, Institut Pasteur, Paris, France

**Keywords:** Harmonin homology domains, Profile HMM, Screening, Phylogeny, Usher syndrome, Sequence analysis

## Abstract

**Background:**

Harmonin Homogy Domains (HHD) are recently identified orphan domains of about 70 residues folded in a compact five alpha-helix bundle that proved to be versatile in terms of function, allowing for direct binding to a partner as well as regulating the affinity and specificity of adjacent domains for their own targets. Adding their small size and rather simple fold, HHDs appear as convenient modules to regulate protein–protein interactions in various biological contexts. Surprisingly, only nine HHDs have been detected in six proteins, mainly expressed in sensory neurons.

**Results:**

Here, we built a profile Hidden Markov Model to screen the entire UniProtKB for new HHD-containing proteins. Every hit was manually annotated, using a clustering approach, confirming that only a few proteins contain HHDs. We report the phylogenetic coverage of each protein and build a phylogenetic tree to trace the evolution of HHDs. We suggest that a HHD ancestor is shared with Paired Amphipathic Helices (PAH) domains, a four-helix bundle partially sharing fold and functional properties. We characterized amino-acid sequences of the various HHDs using pairwise BLASTP scoring coupled with community clustering and manually assessed sequence features among each individual family. These sequence features were analyzed using reported structures as well as homology models to highlight structural motifs underlying HHDs fold. We show that functional divergence is carried out by subtle differences in sequences that automatized approaches failed to detect.

**Conclusions:**

We provide the first HHD databases, including sequences and conservation, phylogenic trees and a list of HHD variants found in the auditory system, which are available for the community. This case study highlights surprising phylogenetic properties found in orphan domains and will assist further studies of HHDs. We unveil the implication of HHDs in their various binding interfaces using conservation across families and a new protein–protein surface predictor. Finally, we discussed the functional consequences of three identified pathogenic HHD variants involved in Hoyeraal-Hreidarsson syndrome and of three newly reported pathogenic variants identified in patients suffering from Usher Syndrome.

**Supplementary information:**

The online version contains supplementary material available at 10.1186/s12859-021-04116-5.

## Background

Hearing relies on the capacity of highly specialized sensory hair cells to transduce sound-induced cochlear vibrations into electrical signals that are transmissible to the brain. Hair cells possess an ensemble of actin-filled stereocilia tightly structured into staircase-shaped bundles deflected by sound-waves. Multiple extracellular links interconnect stereocilia allowing their concerted deflection and thus the simultaneous opening of mechano-transduction channels. Large protein complexes are found at the anchoring sites of these extracellular links. Mutations in the genes encoding these proteins are responsible for hereditary sensory diseases, notably the Usher syndrome [1–4]. This rare disease, affecting the sensory cells of the inner ear and the retina, is the most common genetic cause of combined congenital deafness and progressive blindness, associated in some cases with balance defects [5].

Proteins encoded by usher genes contain numerous scaffolding domains and protein–protein interaction motifs necessary to the intricacy of the network. The molecular interactions mediated by protein domains underlie the cohesive assembly of the complex, but the network’s organization remains elusive. All except one Usher proteins contain short C-terminal motifs that interact with PDZ (PSD95 Dlg1 Zo-1) domains, known as PDZ Binding Motifs or PBM (Additional file 1: Figure S1). In contrast, only three proteins contain PDZ domains and have thus been suggested to act as scaffolds for the assembly of the complexes [6–8], each interacting with several partners. These three scaffolding proteins are Whirlin (DFNB31) [9–11], Harmonin (Ush1C) [12–14] and the PDZ domain containing protein 7 (PDZD7) [15–17], a deafness protein associated with the Usher syndrome. Interestingly, the three proteins also systematically contain protein–protein interaction domains called Harmonin Homology Domain (HHD). This globular domain was first identified in Harmonin and consists of about 70 residues arranged as a bundle of five helices.

**Fig. 1:**
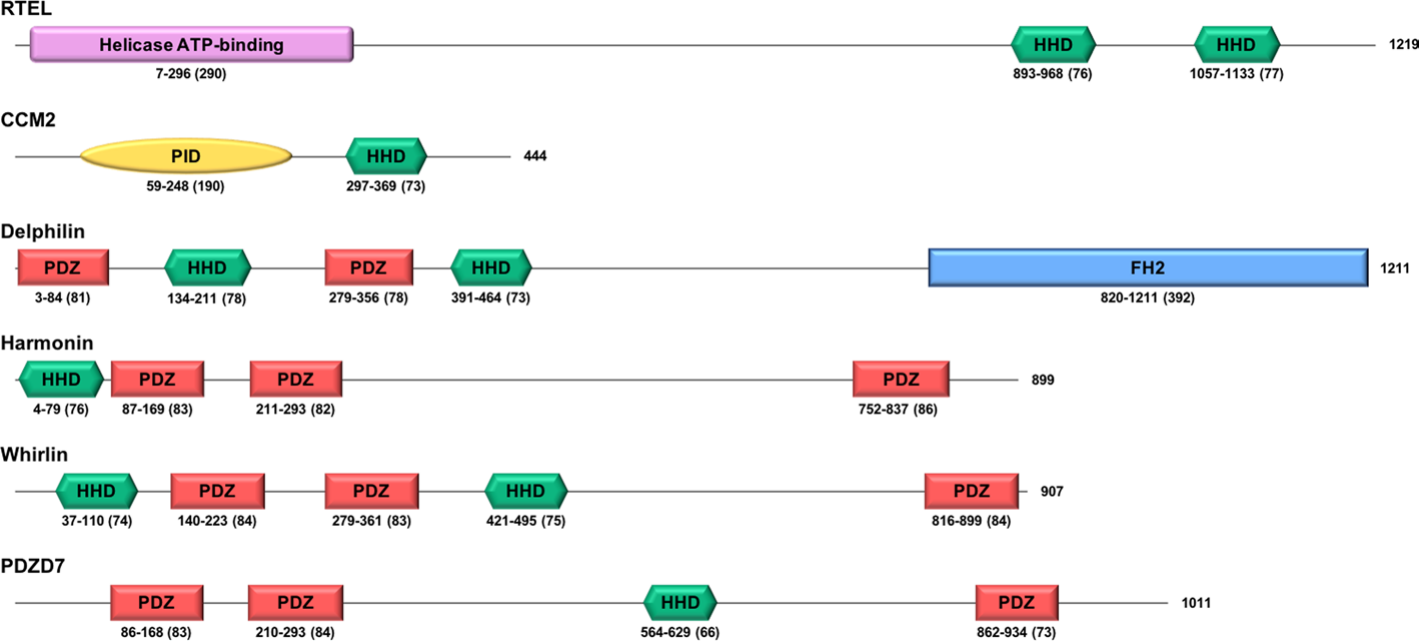
Schematic representation of the six human HHD-containing proteins. Folded domains are highlighted by colored frames. Delimitations of HHDs are derived from our study, delimitations for other domains correspond to the matching UniProtKB entries

Only nine HHDs have been identified in six proteins so far (Fig. 1), including the three Usher-associated and PDZ-containing proteins mentioned before. The post-synaptic scaffolding protein Delphilin (GRID2I) also contains both HHD and PDZ domains. The other two proteins with HHDs are the cerebral cavernous malformations 2 protein (CCM2) and the regulator of telomere elongation helicase (RTEL). The structures of the HHDs from Harmonin and CCM2 proteins have been solved in five hetero-complexes. They exhibit three different binding modes, including inter-molecular [12, 18, 19] and intra-molecular [20, 21] interactions. These structures show that HHDs can bind to isolated amphipathic helices of their partners, but are also prone to interact with larger domains via other surfaces of the alpha-helix bundle, highlighting its plasticity of interaction for binding partners. We previously showed that the intrinsic structural plasticity of the second HHD of Whirlin may play a role in its function, by regulating the interaction with binding partners and promoting its dimerization [22].

Altogether, these results suggest that HHDs are efficient tools to tune protein–protein interactions in rather different biological contexts, through direct binding to the partner and intramolecular modulations of other protein–protein interacting domains, such as PDZ domains, in large modular proteins. In contrast, the variety of proteins in which HHDs are found is surprisingly narrow. Using bioinformatic tools, we have characterized the HHD family diversity and phylogeny from a whole UniProtKB databank screening. We have also reported HHD mutations identified in Usher patients and discussed their functional consequences.

## Results and discussion

### Structure of harmonin homology domains

**Fig. 2:**
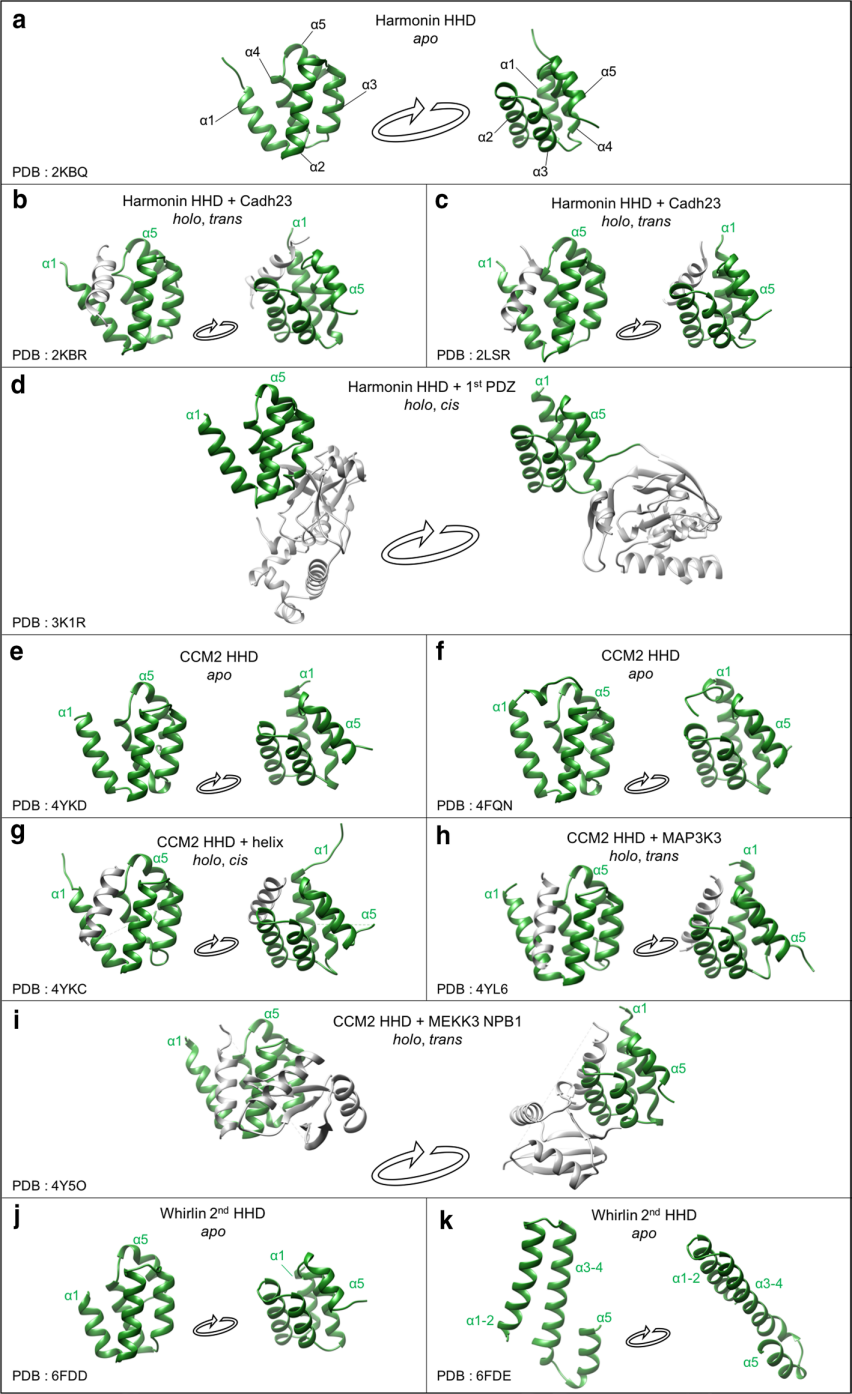
All HHD structures available for Harmonin, CCM2 and Whirlin in the Protein Data Bank (PDB). Structures of free (a, e, f, j, k) and complexed (b, c, d, g, h, i) HHDs. HHDs are colored in green and helical partners or associated domains in gray. The PDB codes are indicated for each structure

Twelve structures of HHDs have been reported so far, covering three out of the nine identified domains and providing information on both free conformations (Fig. 2a, e, f, j, k) and complexes (Fig. 2b, c, d, g, h, i).

As previously described, the free HHDs of CCM2 (PDB entries: 4YKD & 4FQN), Harmonin (PDB entry: 2KBQ) and Whirlin (second HHD, PDB entries: 6FDD & 6FDE) adopt a similar five-helix fold. The main difference concerns the size of the first helix, CCM2 exhibiting the longest (14 amino acids) and the second HHD of Whirlin being the shortest of the three (10 amino acids). The globular fold of the helical bundle is maintained by the extended hydrophobic core of the domain, assisted by essential electrostatic interactions around the inter-helix loops.

The most documented binding mode of HHDs, which can be considered canonical, is reported in Fig. 2b, c, g, h, i. It involves an amphipathic helix from the partner that positions itself between helices α1 and α2 of the HHD. Comparing free (Fig. 2a) and complexed HHDs of Harmonin (Fig. 2b, c) and of CCM2 (Fig. 2e, f against 2g, h), the helix α1 slightly opens upon binding (RMSD values on Ca and N atoms ranging from 1.3 to 3.5 Å), but the rest of the bundle remains mostly identical (RMSD on Ca and N atoms ranging from 0.8 to 1.3 Å). The interaction is primarily driven by hydrophobic effects involving the HHD’s hydrophobic core, but also by hydrogen or ionic bonds between the hydrophilic faces of the helices. As an example, R10 of α1 helix and R31 of α2 helix from Harmonin form ionic bonds with D113 and E111 side chains of Cadherin23, respectively. This mode of binding leads to the interaction in *trans* with the partners (Fig. 2b, c). The binding in *cis* of a helix can also be observed, as illustrated by a structure from CCM2 (Fig. 2i) where a downstream helix from CCM2 folds back to interact with the HHD, competing with the helix from the partner in a regulatory manner [20].

Two other surfaces on the side of the bundle were also observed, providing *cis* (Fig. 2d) and *trans* (Fig. 2i) noncanonical protein–protein interactions, involved in the scaffolding function of HHDs together with the formation of intramolecular supramodules, as illustrated by the HHD-PDZ complex in Harmonin (Fig. 2d) that tunes the binding affinity of the PDZ towards its partners [21, 23].

Finally, HHDs can be found in crystals as oligomers (Fig. 2f, j), or swapped dimer (Fig. 2k) with the merged α2/α3 helices of one monomer interacting with the merged α4/α5 helices of the second monomer [24].

Despite a high structural conservation, available structures suggest that HHDs can accommodate various partners via different binding sites exposed at the surface of the five-helix bundle.

### Identification of HHD-containing sequences

As a first task, we aimed at identifying new HHD-containing proteins. The Harmonin-N-like superfamily from the National Center for Biotechnology Information (NCBI; accession cd07347) was used as a starting set of sequences. It consists of the 94 known sequences of HHDs among the six HHD-containing proteins from various organisms. These domain sequences were used as a seed on which we built a profile HMM (Hidden Markov Model) to screen the UniProtKB database [25, 26]. Profiles HMM are probability-based models allowing to predict true probabilities of finding each residue at every position of an alignment. These are derived from observed frequencies using a statistical approach, instead of directly using these frequencies for scoring. This provides a model that is less biased towards the starting alignment than frequency-based approaches and thus allows to detect more distant homologues. HMM profiles are adequate tools when using a rather short alignment of sequences as a starting point for a large dataset screening.

Screening of the entire UniProtKB database using our generated profile HMM yielded 2939 domain hits, with E-values lower than 10^–5^ as inclusion threshold. Profile HMM and all sequence alignments are accessible online on http://doi.org/10.5281/zenodo.4255847.

**Fig. 3:**
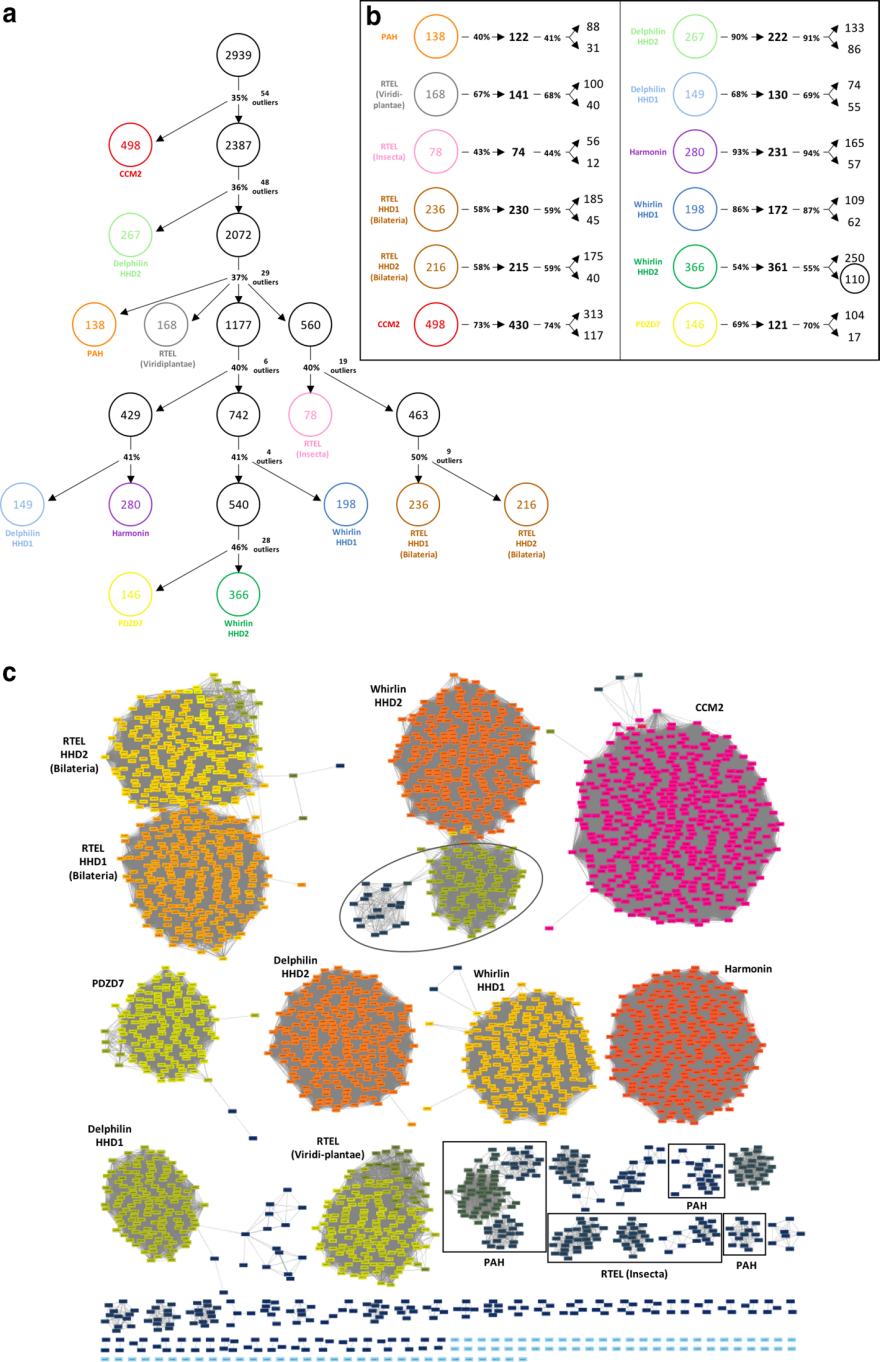
Clustering analysis of HHD-containing sequences. **a** The inclusion threshold (%identity) is incremented from top to bottom. Indicated percentages refer to inclusion thresholds where subclusters emerged, indicated by arrows. Outliers correspond to sequences that did not cluster successfully. The inclusion threshold is further incremented for subclusters with inhomogeneous sequence annotations. **b** Each resulting cluster is again submitted to an incrementation of the inclusion threshold. The first percentage value and arrow correspond to the limit identity where only one main cluster remains and point toward the number of sequences in that cluster. The second percentage value and arrows indicate the identity value where multiple clusters accounting for more than 10% of starting sequences emerge, pointing to the sizes of the two main clusters at the given identity threshold. **c** EFI—Enzyme Similarity Tool representation using a filter-value of 18. This threshold is determined empirically to differentiate and display all clusters in a single snapshot. The circled cluster highlights sequences from insects, corresponding to the Dyschronic sequences also circled in **b**

Our second task was to identify full-length proteins corresponding to each domain hit, given that most associated Uniprot entries were not annotated. We ran the BLASTP algorithm on each domain sequence and corresponding full-length protein, in addition to manually checking each individual UniProtKB entry. This process was supported by a clustering approach (Fig. 3) based on sequence identity of the domains. For this analysis, we started with the whole set of domain sequences (2939) and ran the clustering algorithm with an increasing identity inclusion threshold.

The following procedure was implemented: (1) We increased the inclusion threshold until a significant cluster is isolated, representing more than 5% of the initial full set of sequences. (2) If annotated sequences appear homogeneous, this annotation is used to name the cluster and we proceed to step 3, otherwise we go back to step 1. (3) We further increased the inclusion threshold until another split happened, with hits in subclusters accounting for more than 10% of the total sequences of the parental cluster. The incremental approach allows to isolate all clusters at their appearing threshold. An alternative display of the clustering is presented Fig. 3c. It is the result of a calculation at a single threshold applied to all sequences, thus fragmenting clusters of lower homogeneity (RTEL from insects and PAH).

Our procedure allowed to estimate the conservation within each cluster and to identify subclasses of proteins. From our dataset, only 79 domain sequences (out of 2939) arise from proteins without known HHD or that could not be identified (2.7% of the dataset). These sequences did not cluster together and formed a collection of isolated hits that do not make up for a new HHD-containing family.

Interestingly, 5% of the hits (146 over 2939) corresponded to PAH (Paired Amphipathic Helices) domains that satisfied the inclusion threshold and clustered together. PAH domains are protein–protein interaction domains involved in eukaryotic transcription, found in proteins such as Mad1 and Sin3 [27]. It consists of four helices instead of five as found in HHDs. However, the similarity between PAH and HHDs, considering the four first helices, is striking both in fold and sequence conservation (Faure, et al., Proteins (2014) (https://doi.org/10.1002/prot.24438)) [28], with an overall identity of 12% ± 8%, ranging from 8% between Delphinin-HHD2 and PAH to 17% between HHD from RTEL of plants and PAH (Additional file 1: Figure S2, Table S1). Importantly, PAH also can interact with amphipathic helices through the interface formed by its α1 and α2 helices (ex PDB: 1pd7, 2rms), a binding mode conserved in HHDs. The overall conservation of fold and function suggests the two domains are evolutionary related.

The 2939 sequences are clustered in eight groups shown in Table 1.

**Table 1:** Proteins associated with the 2939 domain hits

Protein name	Number of domain hits	Phylogenetic coverage
PAH-containing	146	Eukaryota
RTEL	787	Eukaryota
CCM2	490	Metazoa
Whirlin	592	Opisthokonta
Delphilin	413	Metazoa
Harmonin	277	Metazoa
PDZD7	155	Metazoa
Unidentified	79	Eukaryota

Their identification was performed using manual blast and clustering results. The number of hits and the smallest phylogenetic group in which the protein is found are indicated for each cluster

#### HHDs in RTEL

We found 787 hits corresponding to HHDs in RTEL proteins from eukaryotic organisms. The HHD is located at the C-terminal of the protein. In contrast with other HHD-containing protein families, HHDs in RTEL form three clusters corresponding to viridiplantae, bilaterian and specifically insects. Based on our analysis, only vertebrates possess two HHDs in their RTEL proteins, as previously suggested [28], whereas a single HHD is found in other bilaterian, insects or plants.

#### HHDs in CCM2

We identified 490 sequences corresponding to HHDs in CCM2 proteins, only found in metazoans. For this family, exclusively one domain hit per protein sequence was found, as previously reported [29]. The HHD domain in CCM2 is identified in the C-terminal 200 amino acid region involved in the induction of cell death.

#### HHDs in Whirlin

We pointed out 592 hits that can be identified as belonging to Whirlin proteins. Two clusters were obtained, one with 198 sequences including the first HHD, and the other with 366 hits including the second HHD of known sequences. However, at an inclusion threshold over 55% identity, this second larger cluster leads to an additional subcluster of 110 hits. Interestingly, these sequences come exclusively from insects, and 75 of them can be identified as a Dyschronic protein, a homolog of Whirlin involved in the synaptic development of flies expressed in major neuronal tracts [30–32]. We can detect only one HHD in these sequences. The N-terminal region of 300 amino acids is predicted as disordered upstream its first PDZ. By contrast, Whirlin has a first HHD in the N-terminal 140 amino acids region upstream its PDZ tandem. The unique HHD of Dyschronic is located between a PDZ tandem, but its HHD is tethered to the third PDZ by a shorter disordered region (about 230 residues instead of 550 in Whirlin). Finally, the Dyschronic protein has 50 additional residues following its third and last PDZ. The two proteins end with a type-I PBM. We hereby predict that the protein referred to as Dyschronic possesses a HHD similar to the second HHD of Whirlin. However, given the confusingly high similarity of Dyschronic to Whirlin, it will be considered as a Whirlin subclass in the rest of the study.

#### HHDs in Delphilin

Among a pool of 413 hits, two clusters were obtained for Delphilin, one comprising the first HHDs and the other the second HHDs of annotated proteins. Protein sequences only originate from metazoans.

#### HHDs in Harmonin

A total of 277 hits correspond to HHDs in Harmonin proteins from metazoans. This cluster has the highest degree of conservation, up to 93% identity for cluster core. The highly conserved residues cover almost all the surface of the HHD. As illustrated in Fig. 2b–d, it has been shown that the HHD of human Harmonin exhibits several surfaces of binding providing both intramolecular (PDZ domain) and intermolecular (Cadherin23) interactions. We suggest that the highest degree of conservation of HHDs in Harmonin might underlie multiple potential binding interfaces, resulting in an overall well conserved surface.

#### HHDs in PDZD7

Coming from metazoans only, 155 hits have been identified as PDZD7 proteins. The vast majority of these sequences encode for one HHD domain, however two HHDs are detected in 11 sequences from 6 organisms (*Branchiostoma floridae*, *Crassostrea gigas*, *Lingula unguis*, *Stylophora pistillata*, *Capitella teleta*, *Strongylocentrotus purpuratus*), with an additional hit before the first PDZ, leading to a Whirlin-like organization of domains. This suggests that PDZD7 might have diverged from Whirlin and lost its first HHD. This observation is consistent with the phylogenetic tree (see below) that highlights a low evolutionary distance between the second HHD of the two proteins.

Overall, only the Dyschronic homologue of Whirlin appears as a new HHD-containing protein. All domain hits identified in this study falls into known HHD-containing protein families, with no significant new architecture. We provide an ensemble of sequences to support HHD studies, with an improved classification of HHD families, highlighting 11 main clusters.

### Phylogeny

**Fig. 4:**
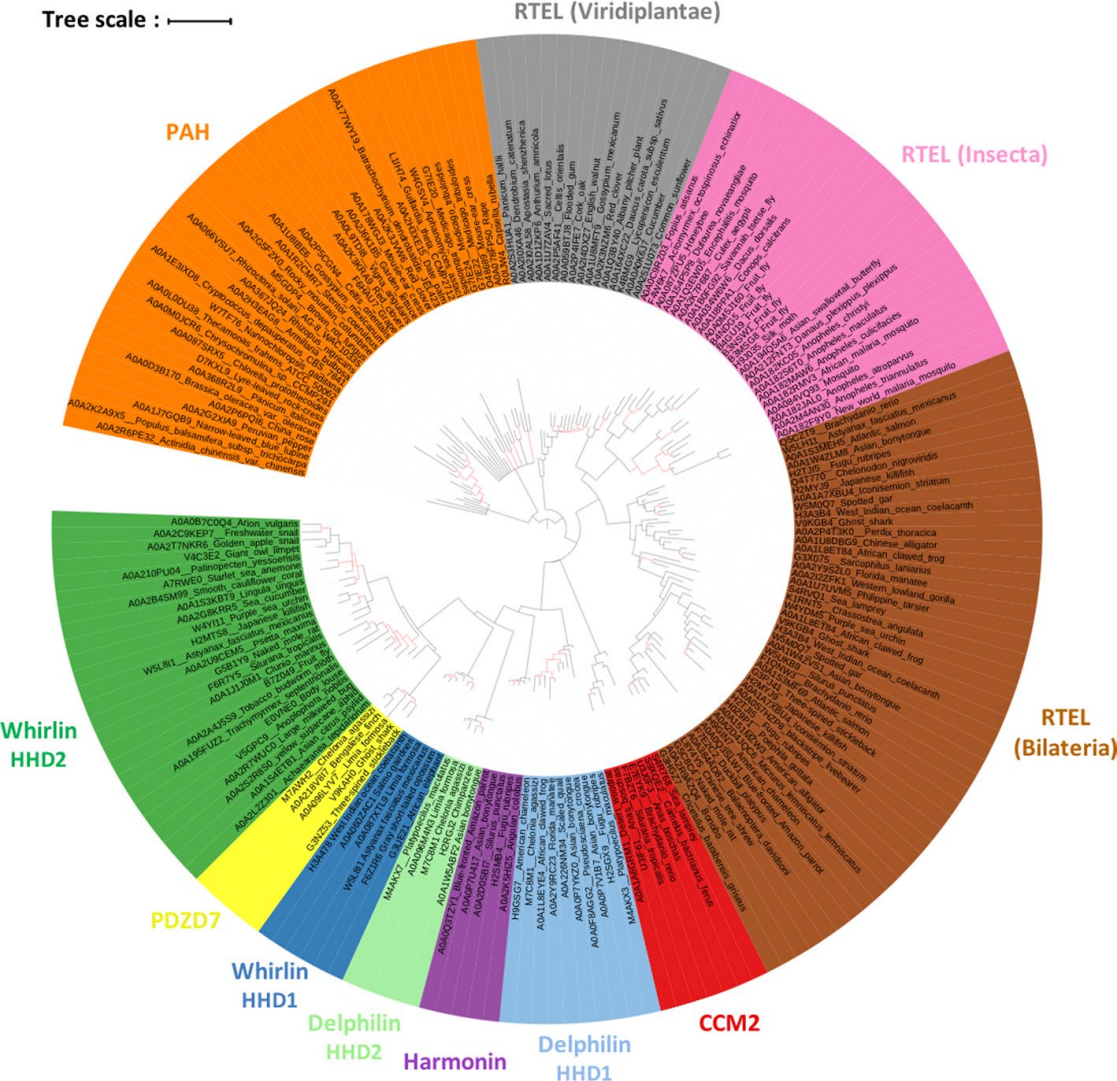
Phylogenetic tree obtained using subsampled sequences from each previously identified cluster. Branches with Transfer Bootstrap Expectation lower than 70% are depicted in red. Phylogenetic tree can be visualized on the iTOL website (https://itol.embl.de/shared/2PuqbCYlUfiSH)

Given the similarity between PAH and HHDs, in terms of conserved residues, fold and canonical binding mode, we hypothesized that PAH and HHDs are evolutionarily related and that the loss or gain of the fifth helix is the evolutionary event to transition from one domain to the other. Using this hypothesis, we decided to root our phylogenetic trees between PAH and HHDs. The various trees we generated based on the full set of sequences presented some variability. This is due to the high number of sequences we compared relatively to their length, resulting in a low signal to anchor each sequence. To overcome this issue, we subsampled each cluster for representative sequences using CD-HIT [33] and successfully obtained a stable tree with statistically supported branching (Fig. 4).

The branching between HHD families in the tree is consistent with the phylogenetic groups in which the proteins are found. First, PAH-containing proteins are found in all eukaryotic organisms and consist in a branch opposed to all HHDs, this is a direct consequence of our working hypothesis. Then, are found HHDs from RTEL in Viridiplantae against all HHDs in Opisthokonta. Among those, a branch containing HHDs from RTEL in Bilateria, with a sub-branching for HHDs in Insecta, is opposed to other HHD containing proteins that are not in plants. Finally, the last branch of the tree exclusively supports neuronal proteins and splits CCM2 from PDZ-containing sequences (Delphilin, Harmonin, Whirlin and PDZD7). The fact that the six HHDs arise closely in the tree suggests that they evolved from one ancestral HHD sequence that was retained only in these neuronal proteins. We note that, at least, the HHD1 domains of Harmonin and Whirlin can form a supramodule organization together with the adjacent PDZ domain that can regulate the inter- and intra-molecular interactions of Usher proteins [19] (also supported by unpublished data). The co-occurrence of HHD and PDZ may indicate a complementarity of function of the domains and suggests that supramodular interactions have been evolutionarily selected in this branch of paralog proteins.

### HHD-family analysis

#### Heatmap

**Fig. 5:**
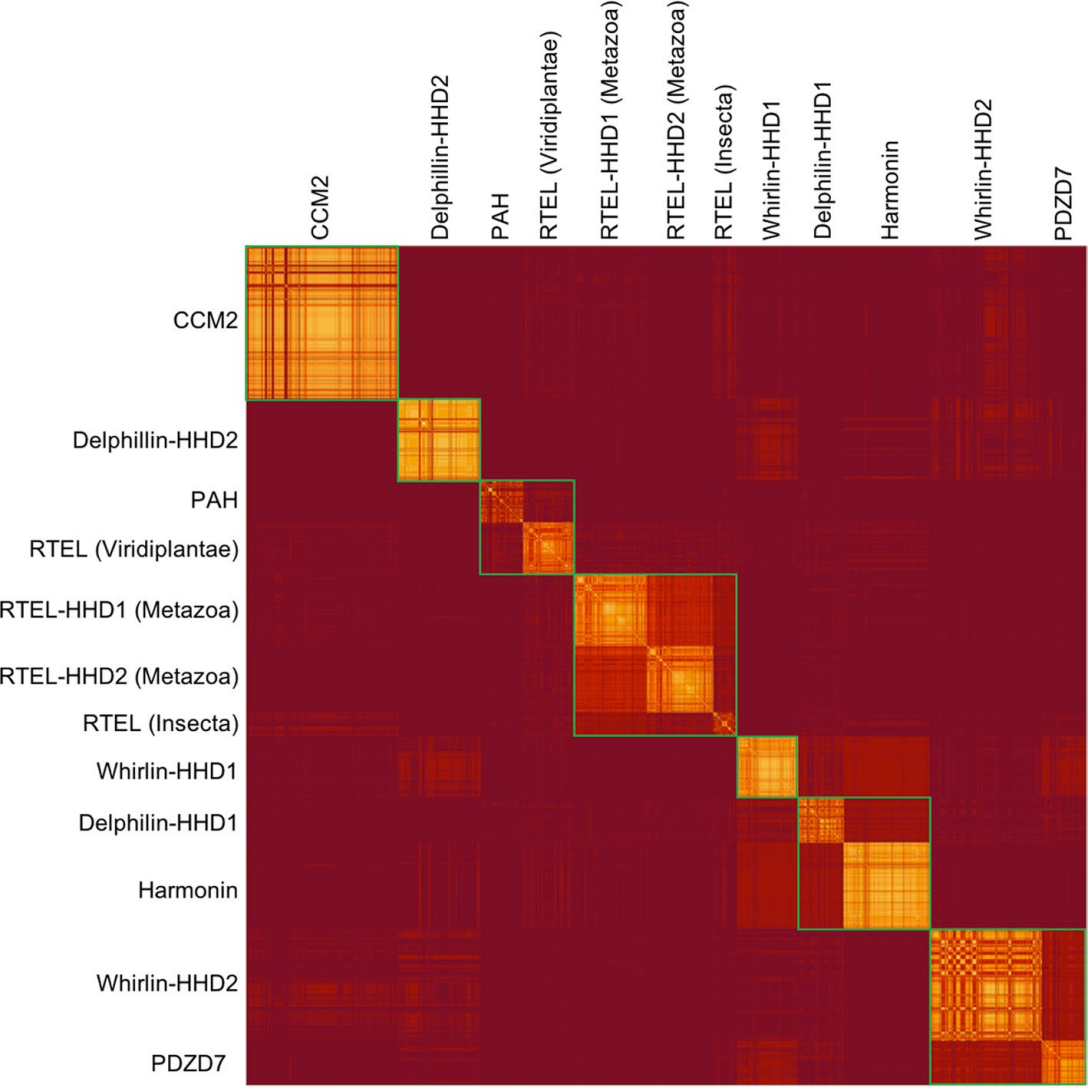
Heatmap based on BLASTp scores - lighter pixels correspond to pairs with a lower e-value, i.e*.* sequences of higher similarity. Communities are indicated by green squares

Phylogenetic analysis based on isolated domains helps to understand the processes promoting protein differentiation, notably through partial (domain) or whole gene (protein) duplication. However, it is not straightforward to conclude on which ensembles of sequences, or branches, are significantly different from one another. Here, we used the BLASTP tool to score each HHD sequence against every other sequence detected using our HMM approach. Converting the obtained data to an adjacency matrix allows us to cluster sequences into communities. Sequences within one community are then not significantly different. Using this approach, we could group all sequences of Whirlin HHD2 and PDZD7 HHD, Delphilin HHD1 and Harmonin HHD, RTEL HHD1 and HHD2 in Metazoa and the specific cluster from insects, as well as the sequences from PAH and HHDs from plant RTEL. CCM2 HHD, Delphilin HHD2 and Whirlin HHD1 are significantly different from any other HHD cluster (Fig. 5).

These results are consistent with the phylogenetic tree (Fig. 4) and branches can be regrouped as follow: PAH and HHDs from RTEL-Viridiplantae; HHD1 and HHD2 in RTEL-Metazoa and in RTEL-Insecta; CCM2 HHD; Delphilin HHD1 and Harmonin HHD; PDZD7 HHD and Whirlin HHD2; Delphilin HHD2; Whirlin HHD1.

#### Motifs analysis

**Fig. 6:**
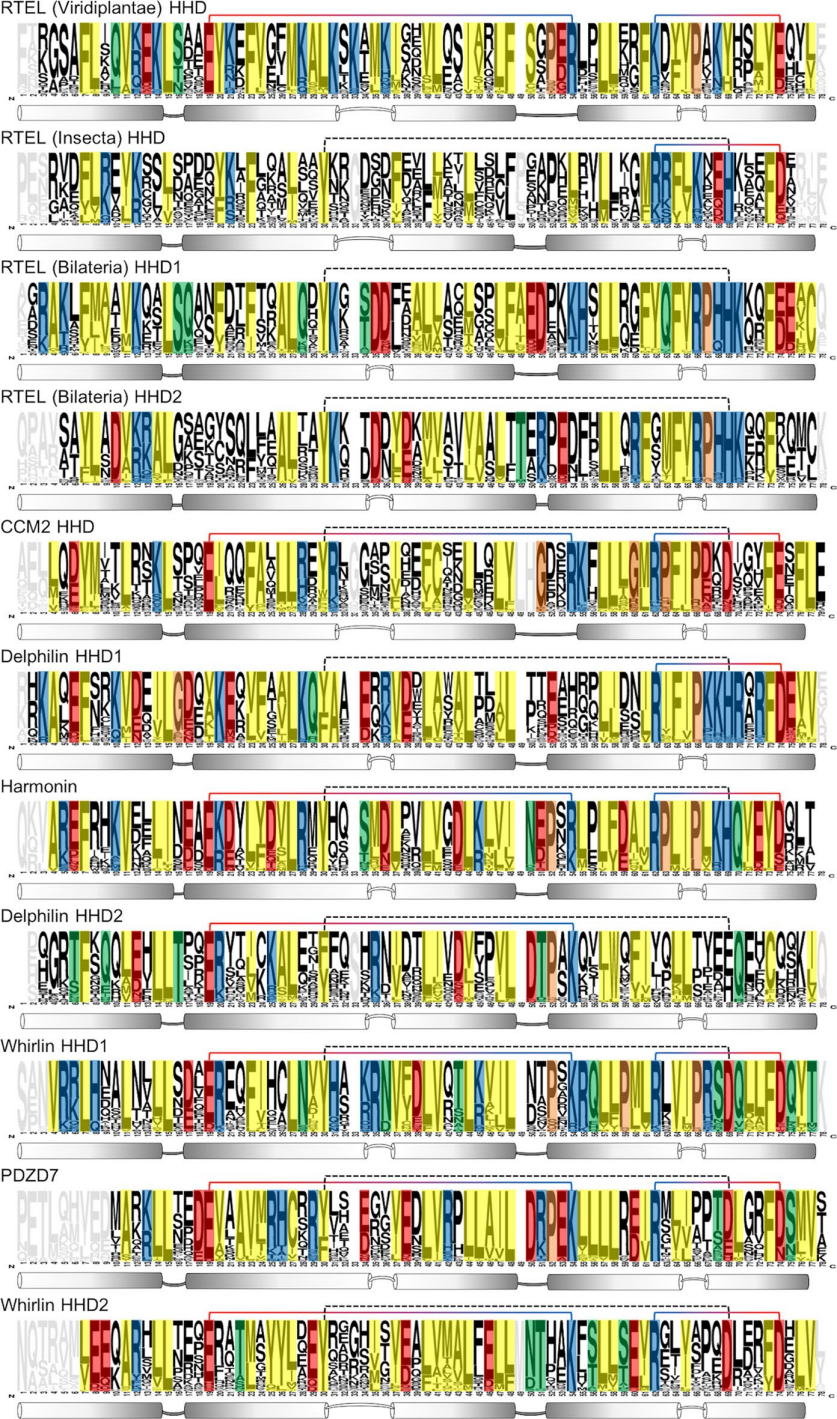
Logo representation of sequences for each individual HHD, extracted from the overall alignment of all HHDs to maintain column correspondence. Clusters corresponding to each HHD were filtered using CD-hit at 95% identity threshold to remove redundant sequences and increase diversity. Number of remaining sequences: 68 RTEL (Viridiplantae), 54 RTEL (Insecta), 83 RTEL HHD1 (Bilateria), 73 RTEL HHD2 (Bilateria), 78 CCM2, 65 Delphilin HHD1, 23 Harmonin, 42 Delphilin HHD2, 33 Whirlin HHD1, 48 PDZD7, 91 Whirlin HHD2. Grayed positions are represented in less than 80% of the sequence hits, non-colored positions are represented in most sequences (>80%), but are not conserved (< 75%) in the final set. Colored positions are conserved in more than 75% of sequences, yellow for hydrophobic, blue for cationic, red for anionic, green for polar and non-charged residues and orange for prolines and glycines. Residues were grouped as follow for conservation calculation: [AVILM], [FY], [TS], [QN], [ED], [RKH], [W], [C], [P], [G]. Recurrent charge-charge and polar interactions are indicated by colored and dashed lines, respectively

Whole domain comparison, as performed with phylogenetic analysis and blast, can fail at differentiating overall similar domains based on short motifs in the sequences. Therefore, the functional features that are specific to each domain, or rather conserved among them, remain elusive. Unfortunately, the use of automatized motif discovery approaches to identify stretches of residues specific to a family, or conserved from one domain to another, did not lead to results we could decipher. This is most likely due to the high conservation within as well as between clusters, leading to a low signal to noise ratio when searching for local stretches of conserved residues with associated functions. Therefore, we manually calculated the conservation for each position of individual HHDs, mapped on a logo representation of the corresponding clusters. Sequences of each cluster were filtered to reduce global redundancy and increase signal for the detection of position-specific conservation. To discuss the conservation of given positions across all HHDs, we will further use the numbering from Fig. 6.

**Fig. 7:**
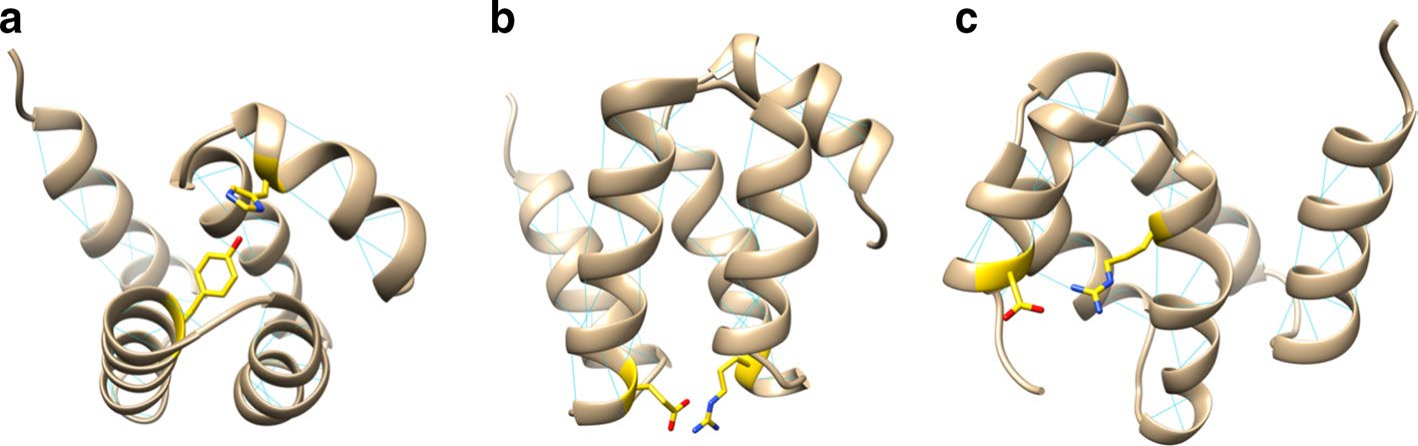
Pair of residues shared among HHDs to promote bundle stability; highlighted on our model of the Harmonin HHD. **a** Positions 30 and 69. **b** Positions 19 and 54. **c** Positions 62 and 74

We identified three stabilizing pairs of positions shared among most HHDs (Fig. 7). The residues position 30 and 69, forming a hydrogen bond (Y to charged) or cation-pi interaction (F to cationic), stabilize the bundle between the top of helices α2 and α5. These two positions are found conserved in all clusters except RTEL from Viridiplantae and the bound is indicated as a dashed line Fig. 6. A second pair of residues, positions 19 (anionic) and 54 (cationic) facing each with charge to charge interaction, stabilizes the bundle between the bottom of helices α2 and α4. The two positions are found conserved in 7 of the 12 clusters as indicated Fig. 6 by a plain colored line. Similarly, a third pair of residues, positions 62 (cationic) and 74 (anionic), links the α4 to the shorter α5, further stabilizing the helix bundle. This last stabilizing pair is found in 8 of the 12 clusters, indicating by a plain colored line Fig. 6.

**Fig. 8:**
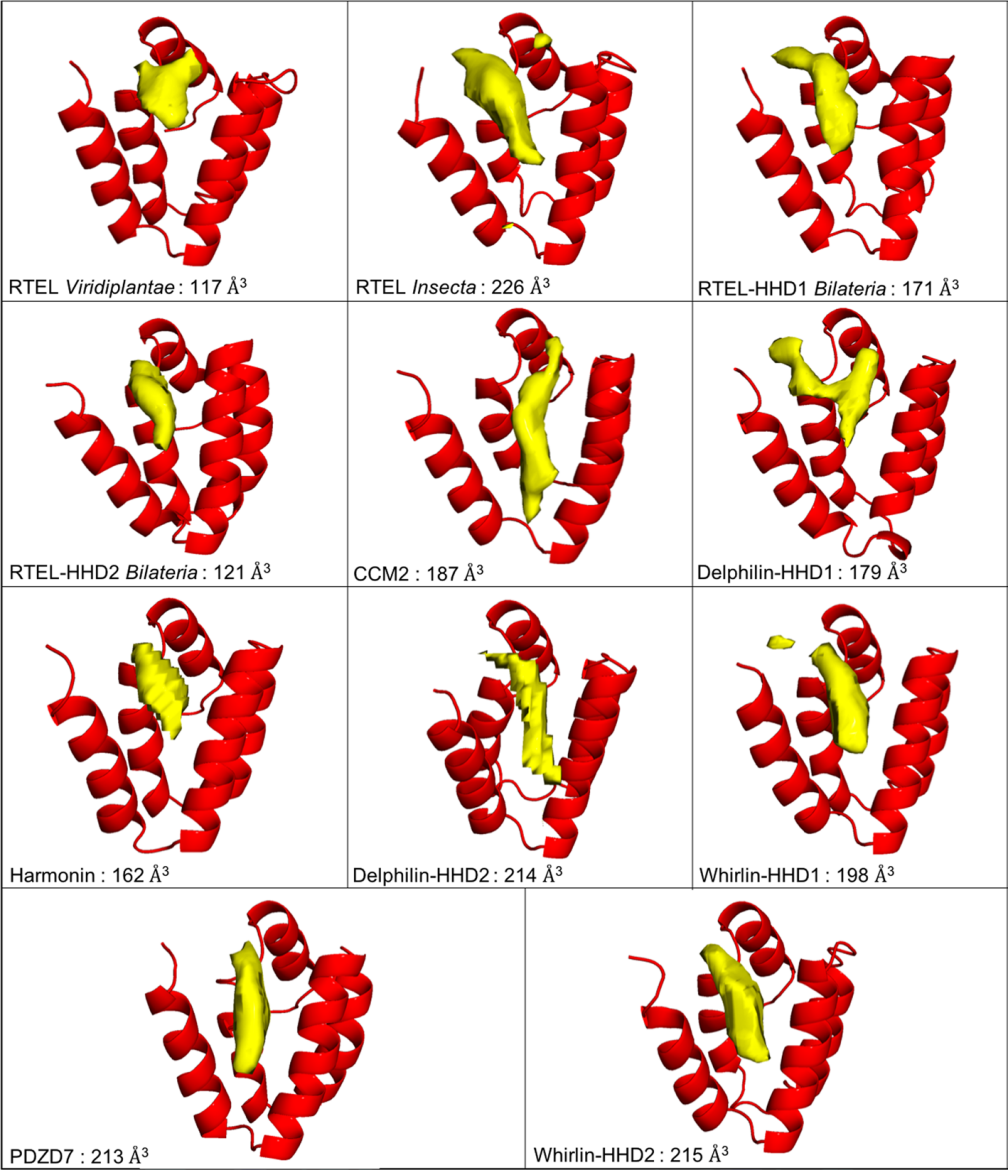
Prediction of protein-protein interaction volumes for each HHD using DeepPPI-Pocket. The predicted volume is depicted at a probability threshold of 37% and its value is indicated for each individual HHD

Based on available experimental models, we identified a list of positions in close proximity to the canonical helix binding site that are or could be involved in the interaction with the partner. They can be sorted into two sets, some with side chain participating in the hydrophobic core of the HHD (positions 7, 11, 27 and 61) forming the background of the binding groove and the others located on the α1 and α2 helices and lining the binding groove (positions 4, 5, 8, 12, 17, 20, 21, 24, 25, 28 and 31). The first set cannot be used to discriminate between HHDs, as the four positions are conserved across all clusters with hydrophobic residues, as taking part in the hydrophobic core of the domain. Looking at the conservation of the second set of positions: 7/11 are conserved in the HHD of RTEL from Viridiplantae, 4/11 for RTEL Insecta, 8/11 for RTEL HHD1 Bilateria, 3/11 for RTEL HHD2 Bilateria, 6/11 for CCM2, 8/11 for Delphilin HHD1, 9/11 for Harmonin, 3/11 for Delphilin HHD2, 8/11 for Whirlin HHD1, 3/11 for PDZD7 and 3/11 for Whirlin HHD2. These observations suggest that some HHDs did not evolve to bind to a helical partner. However, using a protein–protein interaction surface predictor, we could not distinguish the various HHDs in terms of size and shape of the binding site (Fig. 8, method submitted for publication). The binding pocket might be too shallow for accurate prediction, with hydrophobic contacts contributing too much to the interaction compared to subtle variations of residues along the binding site determining their specificity and promiscuity. However, the interface was detected unambiguously for every HHD suggesting that all HHDs could accommodate an amphipathic helix as a binder. More experimental data are required to support any further conclusion.

Two other interfaces for protein–protein interactions are observed in crystal structures. The first one is found on the α2-α3 face of the CCM2 HHD [18] (PDB entry 4Y5O). In the crystal structure, the interaction is mediated by an alpha helix from the MEKK3 protein, as discussed so far, as well as its NPB1 domain, which contacts the HHD on this α2-α3 surface. Picking residues from these two helices facing the NPB1 domain rise the following list of positions: 17, 18, 21, 22, 25, 28, 29, 38, 39, 42, 43 and 46. However, only position 28 is conserved using our criteria, and it is also involved in helix binding. This observation suggests that this interface was not conserved and results from crystal packing, leaving the interaction mainly mediated by the MEKK3 N-terminal helix. This is supported by authors indicating that mutations in the N-terminal helix of MEKK3 severely reduce its ability to pull-down CCM2, while mutations in the NPB1 domain only moderately affect the interaction. The second one consists of the lower segments of α3, α4 and α5 of the Harmonin HHD [21] (PDB entry 3K1R). In the crystal structure, this interface is located at the C-terminus of the HHD and promotes the formation of a supramodule with the following PDZ. Picking positions taking part in this surface of interaction are: 45, 50, 51, 55, 58, 59, 74 and 75. From our study, 7 of the 8 positions are conserved, which is consistent with a functional supramodule affecting the binding of the PDZ to its partners.

### Mutations in HHD domains

According to the low number of HHD-containing proteins and their recent discovery, only few mutations associated with human diseases have been described.

The first HHD of the human RTEL protein is affected by three mutations responsible for the Hoyeraal‐Hreidarsson syndrome (HHS), a severe form of *dyskeratosis congenital* [34]. The first mutation, K897E (UniProtKB entry Q9NZ71), is positioned in the beginning of the first helix (position 5 of the alignment) and is fully exposed to the solvent. This position is conserved in more than 75% of Bilateria RTEL HHD1 sequences we identified in this study. It is located in close proximity to the canonical binding groove of HHDs and the charge switch induced by the mutation could affect the binding of the partner. However, this cannot be ascertained as no helix binding to the RTEL HHD1 have been identified thus far. The second mutation, R957W, is located in the short loop between helices α4 and α5, corresponding to the position 66 of the alignment. This position is conserved and fully exposed and the function of the arginine or effect of the tryptophan is unclear. The last mutation, F964L is located in the middle of the α5, corresponding to the conserved position 73 of the alignment. This phenylalanine takes part in the hydrophobic core of the HHD, occupying a rather large pocket. This mutation might destabilize the core of the domain by introducing a shorter aliphatic side-chain.

Harmonin, Whirlin and PDZD7 are deafness proteins sometimes associated with the Usher syndrome. We have identified 99 variants in HHD domains of these proteins on the largest cohort of USH patients studied to date [35]; 37 in Harmonin HHD, 25 in Whirlin HHD1, 22 in Whirlin HHD2 and 12 in PDZD7 HHD. Three of them have been identified as pathogenic, while the 96 others are classified as Variants of Unknown Significance (VUS, complete list per domain Additional file 1: Table S2). The first pathogenic variant is the R63W mutant in the Harmonin HHD. It corresponds to the conserved arginine position 62 of the alignment, involved in the stability pair stabilizing helices α4 and α5, as previously described. This mutation may thus impair the stability of Harmonin HHD. The second pathogenic variant is the D447H mutant in Whirlin HHD2. This mutation corresponds to position 28 of the alignment and is not conserved. However, this position is located right next to the binding groove and is directly involved in the binding to the helix in Harmonin and CCM2. This mutation may thus affect the binding to a partner. The last pathogenic variant is the R490H mutant in Whirlin HHD2. It corresponds to the non-conserved position 72 of the alignment, completely exposed to the solvent, and its effect is unclear.

## Conclusion

HHDs are found across the whole Eukaryota phylogenetic domain and evolved from a single ancestor, in common with PAH domains. However, only six proteins could be detected in our screening, resulting in a surprisingly narrow variety of proteins encompassing. Adding to these six proteins, we identified a subcluster containing sequences referred to as Dyschronic, the closest homolog to Whirlin in Drosophila. The Dyschronic protein is a component of the circadian output pathway present in the fly central brain. This study led us to identify one HHD domain in this protein similar to the second HHD of Whirlin. Among HHD-containing proteins, the human RTEL protein was reported to possess two HHDs [28]. Here, we highlight that only bilaterians have a two-HHDs RTEL protein, except insects, while RTEL in other organisms only possesses one C-terminal HHD. Interestingly, the HHDs of the various RTEL proteins constitute multiple clusters, reflecting consistent differences in sequence that could be associated with the variability of telomeric properties between taxons.

HHDs are involved in intermolecular interactions, both in homotypic (dimer) and heterotypic (HHD-helix, HHD-PDZ) complexes. The small number of domains identified in the UniProtKB databank, and the diversity of molecular recognition characterized up to now prevent us from predicting potential partners. The main function of HHD is likely to promote the formation of large oligomeric complexes with the recruitment of multi-domain scaffold proteins. The reasons are still unclear to explain why the number of identified HHD is limited despite our extensive search. HHDs are small and well-folded domains and would be good candidates for duplication in proteins to serve as molecular scaffolds for partner interactions. We are currently investigating the role of HHDs in Whirlin and PDZD7 proteins to shed light on the roles of these domains in hearing-associated protein networks. These results will provide clues to decipher the molecular basis of their interactions.

## Methods

### Sequence alignments

All sequence alignments in this paper were performed using the G-INS-i algorithm from the MAFFT package [36], suitable for sequences containing single domains. Position filtering depending on gap percentage was carried out, when indicated, with Goalign “clean sites” option (https://github.com/evolbioinfo/goalign). This allows to remove positions that are not representative of the overall alignment, but rather of unique sequence features.

Alignment quality was assessed using TCS from the T-COFFEE package (http://doi.org/10.1093/molbev/msu117).

### Profile HMM and hits search

Hmmbuild (default parameters) from the package HMMER [37] was used for profile Hidden-Markov Model (profile HMM) building based on sequence alignment. This model consists of probabilities to find a residue at a given position derived from observed counts for each amino acid at this given position, and counts at this position depending on the preceding residue. Hmmsearch from the same package was then used to scan all UniProtKB entries (2018/12/10) for hits with e-values under 10^–5^, regardless of query coverage (–incdomE 1e−5).

### Clustering Analysis and detection of communities

Pairwise all against all blast were performed among the 2939 hits using BLASTp from the BLAST + package [38] (default parameters). Clustering was performed tuning identity cutoff for cluster inclusion using silix [39] (default parameters, only inclusion threshold -i is changed). From the matrix of Blastp scores we extracted a matrix of significant scores by removing all values greater than 1e-4. We took the log of these scores and calculated an adjacency graph using the R package igraph [40]. We then detected the communities in this graph using the Louvain algorithm [41] from the same package. Resulting communities are depicted by green boxes on the heatmap.

### Phylogeny

Prior to tree calculation, we reduced redundancy within the dataset using CD-HIT with a 70% identity threshold on clusters presented Fig. 3b, after identity threshold incrementation (-c 0.7 -b 10 -T 6 -n 4 -d 500 -g 1 -M 2000). The Harmonin cluster (231 sequences) was processed separately with 92% identity threshold to obtain more than one representative sequence. The tree in Fig. 4 was generated using IQ-TREE v1.6.7.2 [42] with automatic extended model selection [43] followed by tree inference. The selected model was LG + R5. We used the option -allnni, set the -pers option to 0.2 and the -nstop criterion to 500 for more thorough searches. The alignment was processed 50 times and only the best tree was retained.

Using the same software, model and search parameters, we performed 1,000 bootstrap inferences resulting in 1,000 bootstrap trees. We used these trees to calculate Transfer Bootstrap Expectation branch support values [44] using Goalign.

### Motifs

The overall alignment of all HHDs detected in our study was filtered to remove columns containing more than 90% gaps. Sequences gapped for more than 20% of the remaining positions were removed. Subsets of sequences corresponding to each individual HHD were then extracted from the overall sequence alignment of all detected HHDs, conserving position numbering from one subset to the other. We finally used CD-hit [33] with a 95% identity threshold to reduce redundancy within each subset. Logos were finally generated using the online tool WebLogo (logo@combio.berkeley.edu) to represent amino acids frequencies at each position of the alignment for the individual HHDs (i.e. subset).

### HHD Modeling

HMM-detected sequences to model are aligned to the HHD sequence of the human Harmonin (UniProtKB: Q9Y6N9), using delimitations from a published *holo* structure (PDB: 2KBR). In order to account for possibly shorter predicted sequences due to an increased variability at domain extremities, flanking regions to the HMM prediction are added as necessary to match template length. Models are calculated using MODELLER [45] using the automodel() function. The best out of 10 generated models (lowest MODELLER objective function value) is retained. For the DeepPPI-Pocket prediction, side chains orientation within the binding groove is optimized using HADDOCK [46] in the presence of the ligand from the template Harmonin structure (PDB: 2KBR).

### Protein–protein interaction surface predictor

In order to make the protein–protein interaction surface prediction between HHD and its partners (Fig. 8), we have used DeepPPI-Pocket (manuscript under preparation), a deep-learning model based on a double-task FCN (Fully Convolutional Network) capable of predicting the interaction sites of other protein partners or the binding sites of small molecules ligands, from the three-dimensional structure of a protein. This artificial neural network has been trained from known structures of protein complexes and small molecule protein–ligand complexes. Thus, the 3D structure of the protein, here HHD, is placed within a 3D grid and probabilities of binding another protein are predicted for each grid point. The combination of the probabilities allows us to measure a volume for the binding site that matches a chosen probability threshold. Once a specific threshold is fixed (here 0.36) the comparison of the volumes between different systems can be used to gauge the propensity of a protein region to bind another protein.

## Supplementary information

**Additional file 1.** Schematic representation of proteins part of the Usher interactome. Metrics derived from the BLAST results between sequences from the PAH cluster and each HHD cluster. All identified Variants of Unknown Significance (VUS) found in the Harmonin, Whirlin and PDZD7 HHDs.

## Data Availability

Phylogenetic tree can be visualized on the iTOL website (https://itol.embl.de/shared/2PuqbCYlUfiSH). Profile HMM and all sequence alignments are accessible online on http://doi.org/10.5281/zenodo.4255847. Any additional information will be provided under request.

## Abbreviations

HHD: Harmonin homology domain
PDZ: PSD95 Dlg1 Zo-1
PAH: paired amphipathic helices
RTEL: regulator of telomere elongation helicase
CCM2: cerebral cavernous malformations 2 protein
PDZD7: PDZ domain containing protein 7
HMM: hidden Markov model
HHS: Hoyeraal−Hreidarsson syndrome
