# Supplementary material for: Phylogenetic analysis of Harmonin homology domains
Source: BMC Bioinformatics. 2021 Apr 14;22:190. doi: 10.1186/s12859-021-04116-5 (PMC8048344; doi:10.1186/s12859-021-04116-5)
Supplement: Supplementary file 1 — Additional file 1. Schematic representation of proteins part of the Usher interactome. Metrics derived from the BLAST results between sequences from the PAH cluster and each HHD cluster. All identified Variants of Unknown Significance (VUS) found in the Harmonin, Whirlin and PDZD7 HHDs. [file 12859_2021_4116_MOESM1_ESM.pdf]

Supplementary Materials

Phylogenetic analysis of Harmonin Homology Domains

Baptiste Colcombet-Cazenave[1,2], Karen Druart[3], Crystel Bonnet[4,5], Christine Petit[4,5], Olivier Spérandio[3], Julien Guglielmini[6], Nicolas Wolff[1*]

1. Unité Récepteurs-Canaux, Institut Pasteur, 75015 Paris, France
2. Sorbonne Université, Collège Doctoral, F-75005 Paris, France
3. Unité de Bio-informatique Structurale, Institut Pasteur, 75015 Paris
4. Unité de Génétique et Physiologie de l'Audition, Institut Pasteur, 75015 Paris, France
5. Institut de l'Audition, Institut Pasteur, INSERM, F-75012 Paris, France.
6. Hub de Bioinformatique et Biostatistique – Département Biologie Computationnelle, Institut Pasteur, USR 3756 CNRS, Paris, France

*Corresponding author. E-mail: nicolas.wolff@pasteur.fr

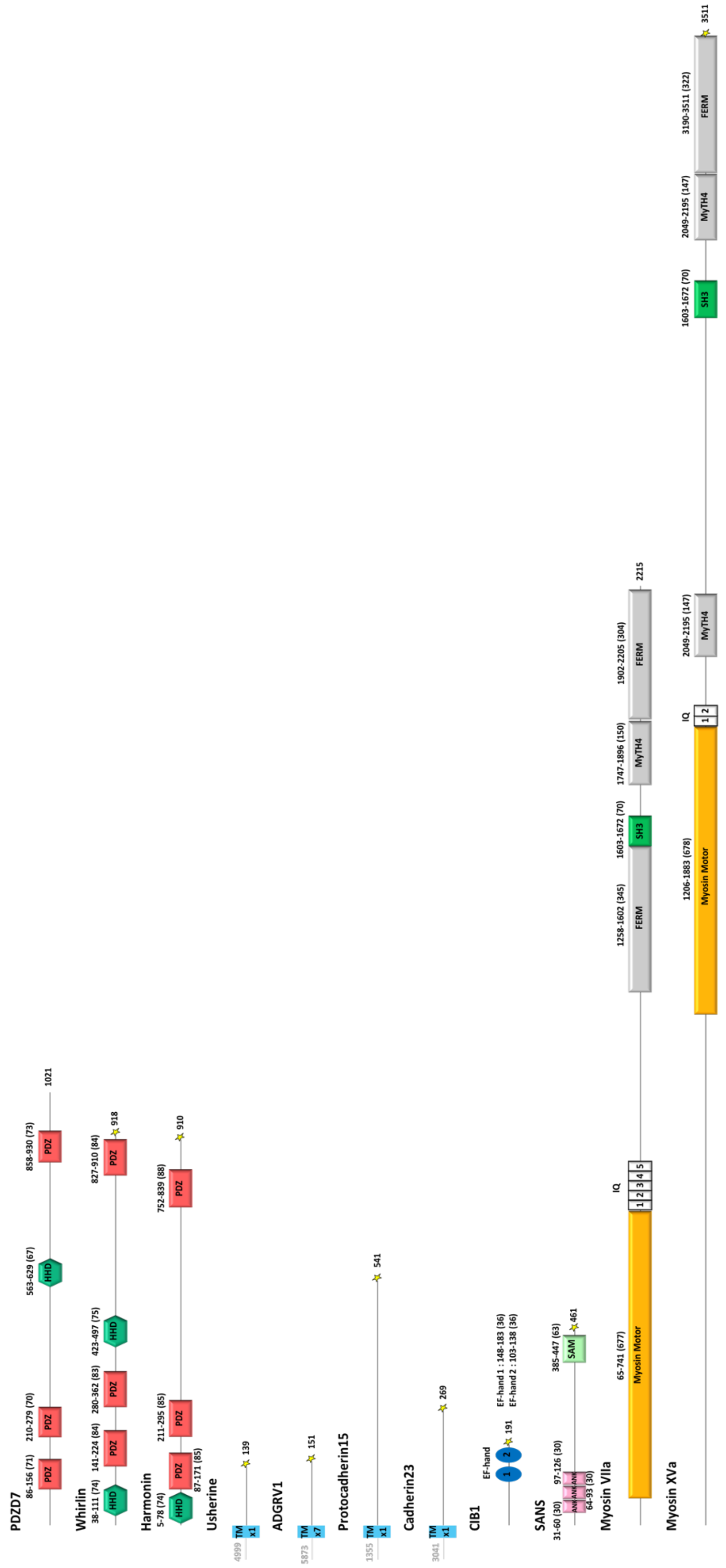

Figure S1: Schematic representation of proteins part of the Usher interactome.

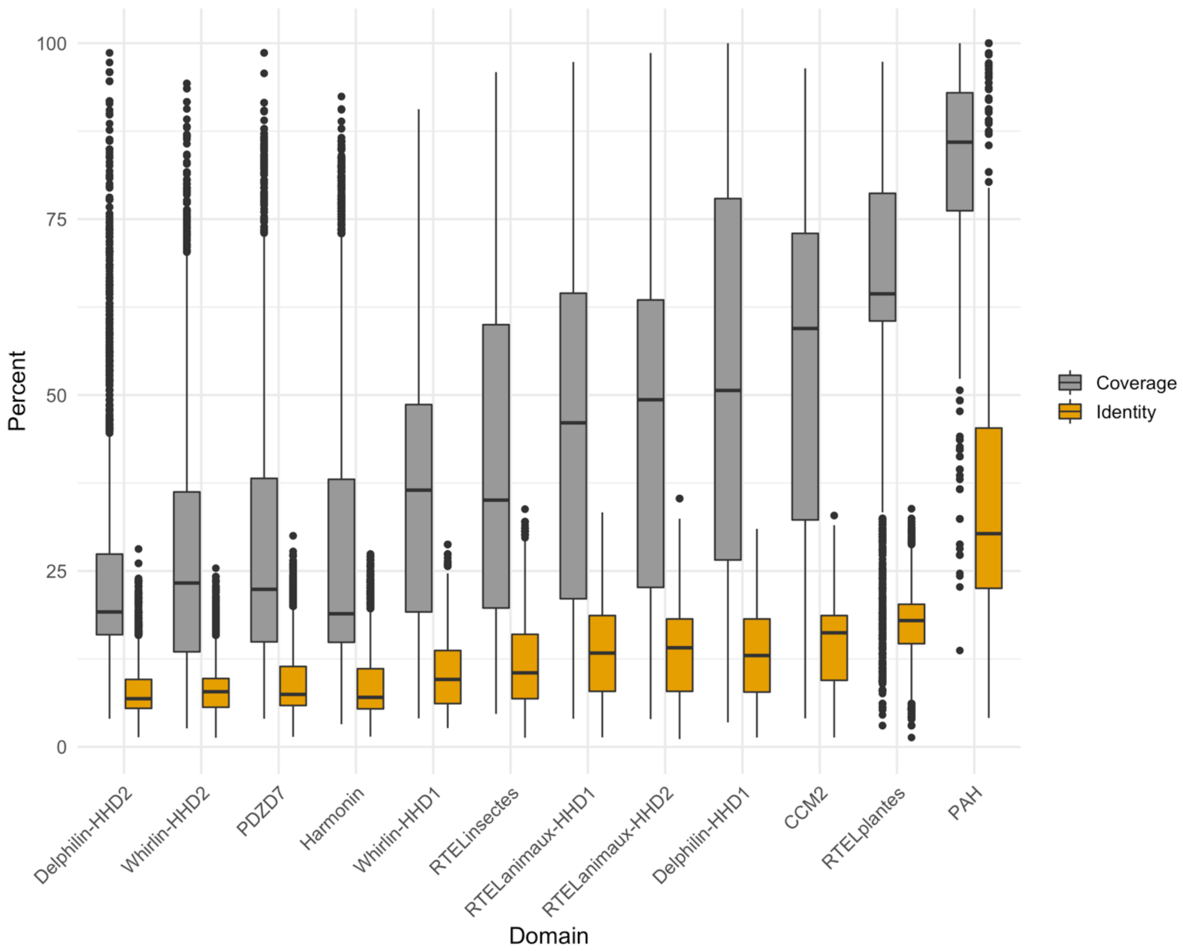

Figure S2: Metrics derived from the BLAST results between sequences from the PAH cluster and each HHD cluster.

Pair	min_id	max_id	mean_id	sdev_id	min_len	max_len	mean_len	sdev_len
CCM2:CCM2	0.0232555	1	0.66957151233586	0.24866273201921	0.075	1	0.92411269925699	0.12389552879798
CCM2:Delphilin-HHD1	0.0256406	0.324677	0.11616529073206	0.047084646012106	0.038961	0.986486	0.37020221533584	0.19768260287627
CCM2:Delphilin-HHD2	0.0133332	0.273972	0.076552256478348	0.038251642395009	0.03125	1	0.23657191311764	0.15922244268714
CCM2:Harmonin	0.0259736	0.317073	0.10902198483219	0.042710391896271	0.0416667	1	0.30287122367721	0.17925433332197
CCM2:PAH	0.013334	0.328768	0.14457515430999	0.054391410751262	0.0405405	0.964286	0.5302542417678	0.24381631295359
CCM2:PDZD7	0.0135135	0.337843	0.13843859658497	0.054492883942006	0.038961	1	0.58206740018636	0.28056897897716
CCM2:RTELanimaux-HHD1	0.0131578	0.305082	0.17732124754909	0.058037963996792	0.0266667	1	0.73072195646484	0.2378093080153
CCM2:RTELanimaux-HHD2	0.0222222	0.28947	0.087200092401086	0.033919051663449	0.0394737	1	0.34039914675089	0.17023537913106
CCM2:RTELinsectes	0.012986	0.36364	0.18169924672794	0.058134457469065	0.0470588	1	0.75362243148105	0.25766848930041
CCM2:RTELplantes	0.0259734	0.319441	0.17624381873984	0.056362157973998	0.038961	1	0.73247737930651	0.25473140823648
CCM2:Whirlin-HHD1	0.0135135	0.328768	0.21236242948815	0.060939919623694	0.027027	1	0.71528802582705	0.21270093427541
CCM2:Whirlin-HHD2	0.0131582	0.355263	0.19877830789524	0.075135847645683	0.0147059	1	0.68005817737178	0.23374336454229
Delphilin-HHD1:Delphilin-HHD1	0.11594	1	0.50309759042264	0.19541123122598	0.373913	1	0.88074698376564	0.11427764249176
Delphilin-HHD1:Delphilin-HHD2	0.0259736	0.310811	0.11810903708619	0.041757595727824	0.0361446	1	0.47244302877988	0.192806132622
Delphilin-HHD1:Harmonin	0.0144923	0.389611	0.23993231471596	0.056018799204549	0.0606061	1	0.81228005058725	0.16208042736295
Delphilin-HHD1:PAH	0.0133328	0.309859	0.13129598734316	0.05738924678514	0.0347826	1	0.51376163019648	0.26149759456849
Delphilin-HHD1:PDZD7	0.0259738	0.356167	0.18481127458398	0.04467107495096	0.0394737	1	0.62230061943551	0.15229367977967
Delphilin-HHD1:RTELanimaux-HHD1	0.025641	0.324322	0.13870989829655	0.050557677656349	0.0347826	1	0.52346939027699	0.21229412263718
Delphilin-HHD1:RTELanimaux-HHD2	0.0129873	0.337843	0.15805643958955	0.055169324409104	0.0384615	1	0.59873721566151	0.22100423756765
Delphilin-HHD1:RTELinsectes	0.0129862	0.319997	0.13225005425916	0.053808766427552	0.038961	0.988764	0.50604536994063	0.23219362834939
Delphilin-HHD1:RTELplantes	0.0131578	0.315789	0.090787118028923	0.045098903882818	0.025641	1	0.35507775310203	0.19735760551746
Delphilin-HHD1:Whirlin-HHD1	0.0384615	0.338028	0.19841680862653	0.039539358242318	0.0519481	1	0.6728232572029	0.13289670854374
Delphilin-HHD1:Whirlin-HHD2	0.0147062	0.364862	0.17176753371933	0.055951909366859	0.0384615	1	0.54207093421352	0.18145004696333
Delphilin-HHD2:Delphilin-HHD2	0.134021	1	0.73786233096787	0.23014850666192	0.360825	1	0.97081042640309	0.054887228975337
Delphilin-HHD2:Harmonin	0.0277779	0.337834	0.17185017275682	0.03855861164981	0.0416667	0.972973	0.78482123483213	0.16441103828842
Delphilin-HHD2:PAH	0.0136981	0.28125	0.079254624686533	0.031833329813251	0.04	0.986301	0.226387491858	0.12035450466712
Delphilin-HHD2:PDZD7	0.038961	0.342857	0.17059049894823	0.036853046837445	0.0416667	0.945205	0.66897777945205	0.12313801126206
Delphilin-HHD2:RTELanimaux-HHD1	0.0131582	0.293333	0.12300119623643	0.043661230979887	0.0394737	1	0.5377572659303	0.23204401509458
Delphilin-HHD2:RTELanimaux-HHD2	0.0206187	0.293333	0.08187633675267	0.034291956116533	0.0266667	0.986667	0.32104222026113	0.16545344487877
Delphilin-HHD2:RTELinsectes	0.0136986	0.28767	0.10472893473783	0.046016896057476	0.0422535	1	0.44063444872035	0.24984501338886
Delphilin-HHD2:RTELplantes	0.0133335	0.319997	0.081118282855136	0.036795968990821	0.0309278	1	0.28963884736379	0.16548692320352
Delphilin-HHD2:Whirlin-HHD1	0.04	0.353848	0.21440287375818	0.038485689913859	0.106667	0.971831	0.80690519520107	0.14123412715611
Delphilin-HHD2:Whirlin-HHD2	0.0138883	0.328951	0.1708985584592	0.051650981880094	0.0410959	0.985507	0.57556134499959	0.15522604622354
Harmonin:Harmonin	0.130433	1	0.78968133719918	0.19927020552792	0.354839	1	0.89696167825141	0.12608286275545
Harmonin:PAH	0.014493	0.273972	0.09483078827381	0.056070666891355	0.0322581	0.924242	0.30231252019798	0.21802714857471
Harmonin:PDZD7	0.0263154	0.314282	0.14156291709638	0.039160492908406	0.0410959	1	0.51602793104697	0.14591602730103
Harmonin:RTELanimaux-HHD1	0.0263154	0.328767	0.13575212510518	0.039603422381635	0.0394737	1	0.64035655248033	0.18230530559819
Harmonin:RTELanimaux-HHD2	0.0222222	0.280003	0.12695020183532	0.051309103220098	0.038961	0.986486	0.49465814628307	0.21432214772544
Harmonin:RTELinsectes	0.0259738	0.285718	0.12218038274954	0.05763823829581	0.04	0.972973	0.53322531981914	0.25340301454253
Harmonin:RTELplantes	0.0259743	0.297301	0.12628272060162	0.040479804509875	0.056338	0.986486	0.5125627135034	0.18782184752982
Harmonin:Whirlin-HHD1	0.0540541	0.34247	0.25049916537789	0.030083793088396	0.108108	1	0.85094670983045	0.10933545786121
Harmonin:Whirlin-HHD2	0.025974	0.309862	0.12541631150517	0.045122969376714	0.04	0.987013	0.49780832271565	0.17016746352882
PAH:PAH	0.0410959	1	0.36673253972813	0.19638935415103	0.136986	1	0.84410739146303	0.11287153454107
PAH:PDZD7	0.0142851	0.299997	0.087095030586162	0.042065598677222	0.04	0.986301	0.27117754767471	0.16206431014634
PAH:RTELanimaux-HHD1	0.013513	0.333334	0.13722560404999	0.066620328483387	0.04	0.973333	0.46214233478107	0.25349322991337
PAH:RTELanimaux-HHD2	0.0111111	0.35294	0.13542421144827	0.059380331945491	0.0394737	0.986111	0.47119219437567	0.24257388437813
PAH:RTELinsectes	0.0129865	0.337834	0.11936094240059	0.060300837181637	0.046875	0.958904	0.39836876178465	0.23709460058902
PAH:RTELplantes	0.0133325	0.338462	0.17355632801285	0.048538997574734	0.030303	0.973684	0.64374163785154	0.1724761907924
PAH:Whirlin-HHD1	0.0266673	0.287668	0.10274344017347	0.043970123373673	0.0405405	0.90625	0.35974573293625	0.17922793423041
PAH:Whirlin-HHD2	0.012987	0.253966	0.081025196040231	0.032217147082973	0.0263158	0.942857	0.26378238745446	0.1516174010296
PDZD7:PDZD7	0.219177	1	0.64041425795938	0.18010573279183	0.403846	1	0.92109065668399	0.097658857760296
PDZD7:RTELanimaux-HHD1	0.0263154	0.302635	0.12894061137828	0.043515816210503	0.0263158	1	0.47966849977362	0.22233305800744
PDZD7:RTELanimaux-HHD2	0.0263155	0.324326	0.13422151422501	0.048402935715547	0.0263158	1	0.47566461803177	0.22862773689951
PDZD7:RTELinsectes	0.0263158	0.314282	0.13256556613102	0.053942428332927	0.0263158	1	0.52197793215666	0.23616665117311
PDZD7:RTELplantes	0.0128204	0.27027	0.090291638484589	0.038985034068546	0.027027	1	0.37359466703156	0.18283168876611
PDZD7:Whirlin-HHD1	0.0410959	0.394362	0.26360050705687	0.047277777610246	0.119403	1	0.71247142631797	0.14205013126288
PDZD7:Whirlin-HHD2	0.0289855	0.447365	0.29658203924882	0.054256600917291	0.057971	1	0.84198041011678	0.14436341844944
RTELanimaux-HHD1:RTELanimaux-HHD1	0.184208	1	0.59828853041832	0.17789698326141	0.364865	1	0.94991947450415	0.082474307462084
RTELanimaux-HHD1:RTELanimaux-HHD2	0.0777778	0.486842	0.32349512498921	0.05452159037147	0.188889	1	0.89512197315403	0.087410901588913
RTELanimaux-HHD1:RTELinsectes	0.0447763	0.394737	0.25172758184757	0.045076367472097	0.131579	1	0.87307041055519	0.11201399912293
RTELanimaux-HHD1:RTELplantes	0.0263158	0.350649	0.19373733950262	0.058231758550545	0.0519481	1	0.76782723901458	0.2174597133986
RTELanimaux-HHD1:Whirlin-HHD1	0.0263154	0.279999	0.15475311332499	0.064365770593012	0.0394737	0.973333	0.56153244478792	0.21725561810118
RTELanimaux-HHD1:Whirlin-HHD2	0.0129871	0.315791	0.14099368323956	0.062564003392343	0.0263158	1	0.50674241720096	0.25473569544774
RTELanimaux-HHD2:RTELanimaux-HHD2	0.161765	1	0.5626151250646	0.19297930995244	0.5	1	0.93446739308786	0.065390476197688
RTELanimaux-HHD2:RTELinsectes	0.0266667	0.378377	0.2048724860844	0.054786592928034	0.0533333	1	0.79456971183523	0.18868687612523
RTELanimaux-HHD2:RTELplantes	0.0222222	0.352117	0.20070956680996	0.062245474902213	0.0422535	1	0.73112595056493	0.23779291431655
RTELanimaux-HHD2:Whirlin-HHD1	0.025	0.289471	0.10109820652357	0.040950042602065	0.0333333	1	0.33743744287201	0.19772199494488
RTELanimaux-HHD2:Whirlin-HHD2	0.0131582	0.302627	0.10515401148869	0.050110984043767	0.0266667	1	0.34359683109505	0.19932681181887
RTELinsectes:RTELinsectes	0.0705885	1	0.35569928331668	0.16517181458182	0.305882	1	0.91553908341658	0.092150229531694
RTELinsectes:RTELplantes	0.0259732	0.359995	0.16702289805021	0.061798705499872	0.0533333	1	0.69075510045024	0.268564062953
RTELinsectes:Whirlin-HHD1	0.026666	0.287669	0.14923173220992	0.053071081663326	0.04	0.972222	0.58993329180588	0.23841100845408
RTELinsectes:Whirlin-HHD2	0.0131579	0.319998	0.11326915198613	0.059274991960804	0.0394737	0.987013	0.42476558394458	0.25886802085376
RTELplantes:RTELplantes	0.186666	1	0.53446407499287	0.15007606519702	0.551282	1	0.88618154476761	0.10995400991191
RTELplantes:Whirlin-HHD1	0.0259736	0.263887	0.15336502412217	0.049087583887355	0.0266667	0.985075	0.66314799342082	0.23067115994809
RTELplantes:Whirlin-HHD2	0.0128215	0.338031	0.11632178230142	0.043842197749353	0.025974	1	0.5719329991787	0.24204015582638
Whirlin-HHD1:Whirlin-HHD1	0.202704	1	0.78300790229708	0.17314947805333	0.345455	1	0.9648676725632	0.078152515571325
Whirlin-HHD1:Whirlin-HHD2	0.0263158	0.352945	0.23605650367266	0.047483290122615	0.0405405	1	0.73273776260418	0.13808307455057
Whirlin-HHD2:Whirlin-HHD2	0.138891	1	0.55987816801407	0.21754290724595	0.346154	1	0.92953713820645	0.092136615470614

Table S1: Detailed metrics derived from the BLAST results for all cluster pairs.

Harmonin HHD	Whirlin HHD1	Whirlin HHD2	PDZD7 HHD
V5G	A37D	R423P	L564P
V13A	A37V	Q429R	T570A
D14G	N38S	R431W	T578P
A21S	L42P	H439L	R579H
E22G	T47A	M442V	R583Q
D24E	Y65H	Y444C	Y584S
Y25H	R68C	Y445H	H586Q
Y25C	F72I	Y445C	L598M
L26F	F72L	R450C	P604Q
D28N	V75L	R450H	R614T
R31G	T77P	V456L	M628T
R31Q	T77I	A458T	V629M
M32T	V80A	A462V	
Q35H	R89H	T469P	
M37T	P92S	A471G	
V39M	M93T	P473S	
V41M	R95G	L475P	
L42F	R95L	S477P	
V43M	P99R	I483V	
D45A	R100C	R490L	
D45E	S101C	D492N	
L48V	D102Y	D492H	
V49L	L105P		
N51S	D107Q		
E52D	T110A		
S54R			
A55C			
L56M			
I62V			
R63Q			
I66S			
R69Q			
H70Y			
V72M			
D75E			
L77Q			
T78N			

Table S2: All identified Variants of Unknown Significance (VUS) found in the Harmonin, Whirlin and PDZD7 HHDs.
